# Ammonia-lowering activities and carbamoyl phosphate synthetase 1 (Cps1) induction mechanism of a natural flavonoid

**DOI:** 10.1186/s12986-015-0020-7

**Published:** 2015-06-09

**Authors:** Kazunari Nohara, Youngmin Shin, Noheon Park, Kwon Jeong, Baokun He, Nobuya Koike, Seung-Hee Yoo, Zheng Chen

**Affiliations:** Department of Biochemistry and Molecular Biology, The University of Texas Health Science Center at Houston, 6431 Fannin Street, MSB 6.200, Houston, TX 77030 USA; Department of Neuroscience, The University of Texas Southwestern Medical Center, Dallas, TX 75390 USA; Department of Physiology and Systems Bioscience, Kyoto Prefectural University of Medicine, Kyoto, 602-8566 Japan

**Keywords:** Ammonia, Urea cycle, Circadian clock, Diet, Flavonoid, C/EBP

## Abstract

**Objective:**

Ammonia detoxification is essential for physiological well-being, and the urea cycle in liver plays a predominant role in ammonia disposal. Nobiletin (NOB), a natural dietary flavonoid, is known to exhibit various physiological efficacies. In the current study, we investigated a potential role of NOB in ammonia control and the underlying cellular mechanism.

**Materials/methods:**

C57BL/6 mice were fed with regular chow (RC), high-fat (HFD) or high-protein diet (HPD) and treated with either vehicle or NOB. Serum and/or urine levels of ammonia and urea were measured. Liver expression of genes encoding urea cycle enzymes and C/EBP transcription factors was determined over the circadian cycle. Luciferase reporter assays were carried out to investigate function of CCAAT consensus elements on the carbamoyl phosphate synthetase (*Cps1*) gene promoter. A circadian clock-deficient mouse mutant, *Clock*^*Δ19/Δ19*^, was utilized to examine a requisite role of the circadian clock in mediating NOB induction of *Cps1*.

**Results:**

NOB was able to lower serum ammonia levels in mice fed with RC, HFD or HPD. Compared with RC, HFD repressed the mRNA and protein expression of *Cps1*, encoding the rate-limiting enzyme of the urea cycle. Interestingly, NOB rescued CPS1 protein levels under the HFD condition via induction of the transcription factors C/EBPα and C/EBPβ. Expression of other urea cycle genes was also decreased by HFD relative to RC and again restored by NOB to varying degrees, which, in conjunction with *Cps1* promoter reporter analysis, suggested a C/EBP-dependent mechanism for the co-induction of urea cycle genes by NOB. In comparison, HPD markedly increased CPS1 levels relative to RC, yet NOB did not further enrich CPS1 to a significant extent. Using the circadian mouse mutant *Clock*^*Δ19/Δ19*^, we also showed that a functional circadian clock, known to modulate C/EBP and CPS1 expression, was required for NOB induction of CPS1 under the HFD condition.

**Conclusion:**

NOB, a dietary flavonoid, exhibits a broad activity in ammonia control across varying diets, and regulates urea cycle function via C/EBP-and clock-dependent regulatory mechanisms.

**Electronic supplementary material:**

The online version of this article (doi:10.1186/s12986-015-0020-7) contains supplementary material, which is available to authorized users.

## Introduction

Ammonia detoxification is an essential bodily function required for nitrogen homeostasis and physiological well-being [1–3]. Ammonia molecules are produced mainly through catabolism of amino acid and other nitrogenous metabolites in tissues, and also via amino acid deamination and urea salvage by gut bacteria [1, 4]. Whereas a small portion of ammonia is removed via direct renal excretion, the urea cycle in the liver plays a predominant role in ammonia disposal, converting ammonia to the relatively harmless urea for excretion [5, 6]. The urea cycle consists of five enzymatic reactions sequentially taking place in the mitochondrial matrix and the cytoplasm of periportal hepatocytes [7, 8]. Carbamoyl phosphate synthetase 1 (CPS1) catalyzes the first reaction wherein ammonia and bicarbonate combine to form carbamoyl phosphate, and mice deficient in *Cps1* suffered pronounced hyperammonemia and neonatal lethality [9]. As a strikingly abundant protein comprising as much as 20 % of total mitochondrial matrix protein mass [10], CPS1 is also subjected to diverse molecular and cellular regulation. Besides the classically known allosteric activator N-acetylglutamate (NAG), CPS1 level and activity are also regulated by complex molecular and physiological mechanisms [7, 11–16]. Furthermore, coordinate induction of urea cycle components has also been reported at transcriptional and post-transcriptional levels [7, 17].

An emerging cellular mechanism for nitrogen homeostasis is our intrinsic biological timer, the circadian clock [18, 19]. In mammals, core clock genes form interlocked feedback loops, driving gene expression to regulate various metabolic pathways [20–22]. Several earlier studies have implicated a role of the clock in protein or nitrogen metabolism [23–25]. More recently, it was shown that the transcription factor Klf15 is encoded by a clock-controlled gene, and that Klf15 maintains nitrogen balance via regulation of the second urea cycle gene *Otc* [19]. Furthermore, genomic and proteomic studies have also demonstrated circadian variation of *Cps1* mRNA and protein expression in mouse liver, although discrepancies remain concerning mRNA oscillation and protein peaks [26–29].

Apart from the clock being an intrinsic regulator, diet is an external factor that profoundly influences nitrogen homeostasis and ammonia metabolism. Dietary management for genetic hyperammonemia, mainly including protein restriction and supplementation of urea cycle substrate, serves to attenuate ammonia generation and/or bolster ammonia disposal [3, 30–32]. On the other hand, dietary challenges also strongly influence ammonia metabolism and detoxification. For example, the ureagenic capacity of the urea cycle, while far exceeding demand under normal conditions [14], has been shown to be up-regulated in response to greater metabolic challenge [7, 33]. However, ammonia control under varying diet conditions is not well characterized, and little is known regarding functional dietary components for ammonia control besides metabolic substrates [30, 34]. Here, we report novel ammonia-lowering activities and clock-dependent mechanism for urea cycle regulation of a natural polyphenolic flavonoid, Nobiletin (NOB).

## Materials and methods

### Animals

All animal husbandry and experimental procedures were carried out in accordance with approved IACUC guidelines and animal protocols by the University of Texas Health Science Center at Houston (UTHealth). All mice, male and on the C57BL/6 background, were group-housed with 2–4 animals/cage under 12:12 light/dark (12:12 LD) cycles or constant darkness (DD) when indicated. The point of light-on is considered as Zeitgeber Time (ZT) 0, whereas the onset of subjective day during DD is considered as Circadian Time (CT) 0. Animals were *ad libitum* fed with regular chow (RC; LabDiet 5001), high-fat diet (HFD; Research Diets D12492) or high-protein diet (HPD; Research Diets D04080301). Nobiletin was obtained from commercial sources including Sigma and Selleck, and administered via oral gavage (200 mg/kg in 0.5 % Sodium carboxymethyl cellulose) every other day.

### Ammonia and urea assays

Blood and urine samples were collected at ZT6 and ZT18. For determination of serum ammonia and urea concentrations, we employed assay kits (Sigma, AA0100 and MAK006, respectively) according to the manufacturer’s protocols.

### Plasmids

The proximal *Cps1* enhancer [16] was cloned into pGL3 Basic (Promega) using C57BL6/J mouse genomic DNA as PCR template with pF and pR primers (Additional file 1: Table S1). Site-directed PCR mutagenesis was performed to introduce mutations on the C/EBP binding site using P-C/EBPmutF and P-C/EBPmutR primers (Additional file 1: Table S1). Likewise, the distal enhancer [35] was cloned into pGL3 Basic using dF and dR primers. Three C/EBP binding sites in the distal enhancer region were incorporated by DNA synthesis (GeneScript). C/EBPα expression vector was purchased from Addgene (ID12550).

### Luciferase reporter assays

For the luminescence assay, Hepa1-6 cells (ATCC, CRL-1830) were plated the day before transfection at 2 × 10^4^ cells per well in 96-well plates. Cells were transfected with the indicated vectors with *Renilla* luciferase vector for internal control. Twenty four hours after transfection, media was changed to recording media as previously described [36] and treated with different concentrations of Nobiletin. Sealed cultures were placed in an EnVision microplate reader (Perkin Elmer) and bioluminescence from the tissue was recorded.

### Real-time qPCR and Western blot analyses

Total RNAs purified by using Trizol were used for cDNA synthesis and real-time qPCR was performed with an Agilent MaxPro3000 Thermocycler using SyBR green reaction mix (GenDEPOT). The qPCR primers used are listed in Additional file 1: Table S2.

Tissue collection and Western blotting was performed as described previously [37, 38]. Briefly, liver tissue samples were dissected from the same lobular regions in different animals to ensure experimental consistency. The samples were immediately frozen in liquid nitrogen and stored at −80 °C prior to use. The harvested liver tissues were homogenized in extraction buffer containing 0.1 % TritonX-100. Protein samples were separated by 12 or 6 % SDS-polyacrylamide gel then transferred to a nitrocellulose membrane. Anti-CPS1 (Abcam), anti-C/EBPα (Cell Signaling), anti-C/EBPβ (Abcam) antibodies were used. Quantitation of Western blot results was carried out by using ImageJ software (NIH).

### Immunohistochemistry

Liver tissues (right lateral lobe) were collected at the indicated times and immediately fixed in 10 % buffered formalin. For immunohistochemistry, 20-μm sections were collected using a VibroSlice microtome (World Precision Instruments) and processed free floating. Sections were incubated with rabbit anti-CPS1 (1:10,000; Abcam), followed by Alexa Fluor 546 rabbit secondary antibody (1:1000; Invitrogen). After 30 min of DAPI staining (5 μM), Vectashield mounting media (Vector Labs) was used to mount the liver slices. Section images were acquired and analyzed by using a Nikon A1R confocal microscope.

### SDS-PAGE and mass spectrometry

Liver extracts were separated on SDS-PAGE gels and minimally stained with Coomassie blue. The MW 164KD band was excised and digested with trypsin. The sample was subjected to mass spectrometry protein ID at the BCM Proteome Core Facility using a Velos-Orbitrap mass spectrometer. Spectral data were then identified by using Proteome Discoverer Suites with Mascot (Orbitrap data) software [39].

### Statistical analysis

Unless otherwise stated, results are presented as mean ± SEM. Data were analyzed using Student’s *t*-test, one-way ANOVA followed by *post-hoc* analysis using Dunnett’s multiple comparison tests or two-way ANOVA followed by *post-hoc* analysis using Bonferroni test as appropriate. A value of *p* < 0.05 was considered statistically significant.

## Results

### NOB improves ammonium detoxification

**Fig. 1:**
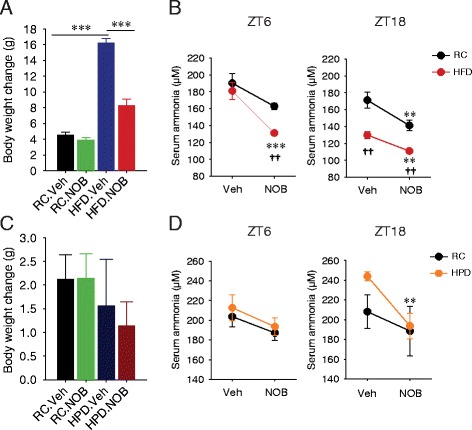
Nobiletin (NOB) lowers serum ammonia levels. **a** Body weight change of wild-type (WT) mice fed with regular chow (RC) or high-fat diet (HFD) and treated with either vehicle (Veh) or Nobiletin (NOB) for 10 weeks (*n* = 11–12). ***p < 0.001. **b** Serum ammonia levels at Zeitgeber Time (ZT) 6 and 18 in the mice described in (**a**). ZT0 corresponds to the onset of light phase. **c** Body weight change of WT mice fed with RC or high-protein diet (HPD) and treated with either Veh or NOB for 4 weeks (*n* = 6–7). **d** Serum ammonia levels at ZT6 and ZT18 in the mice described in (**c**). Data are presented as mean ± SEM. For panels **b** and **d**, Student’s *t*-test: Veh vs. NOB, **p < 0.01, ***p < 0.001; RC vs. HFD or HPD, ††*p* < 0.01

Among a growing number of bioactive polyphenols, Nobiletin (NOB) is a citrus-derived dietary flavonoid with diverse physiological functions [40–43]. More recently, NOB has been shown to display a protective role against high-fat diet (HFD)-induced obesity and other metabolic dysfunctions [44–46]. To investigate a potential role of NOB in nitrogen homeostasis, we examined serum ammonium levels in wild-type (WT) mice under different diets. Under RC, NOB did not significantly affect body weight (Fig. 1a). Consistent with previous findings [44, 45], NOB ameliorated body weight gain under HFD (Fig. 1a). Importantly however, under both diets, NOB markedly reduced serum ammonium levels (Fig. 1b). At Zeitgeber Time (ZT) 6 corresponding to mid-day (inactive phase), NOB led to a more pronounced reduction for HFD than RC (27.5 % vs 14.4 %) (Fig. 1b, left). Ammonia-lowering activities of NOB were also observed at ZT18 corresponding to mid-night when mice were active (Fig. 1b, right). Whereas attenuated serum ammonia levels were observed in HFD.Veh than RC.Veh, likely due to lower protein content in HFD than RC (20 % vs. 29 % in calories), NOB treatment diminished the ammonia levels in both diets by similar degrees.

We next investigated effects of NOB under high-protein diet (HPD), corresponding to enhanced protein metabolism and ammonia flux [19]. NOB did not significantly affect body weight in these mice (Fig. 1c). At ZT6, serum ammonia levels were similar between RC and HPD, and NOB showed a modest trend of reducing serum ammonia levels (Fig. 1d, left). Importantly, during the active phase at ZT18 when serum ammonia levels were significantly elevated in HPD, NOB robustly reduced serum ammonia to RC levels (Fig. 1d, right). Together, these results indicated a broad role of NOB in reducing serum ammonia content at both active and inactive circadian phases under RC, HFD and HPD conditions. Furthermore, NOB more strongly reduced serum ammonia levels under HFD and HPD at ZT6 and ZT18 respectively, serving to normalize the exaggerated circadian variation between active and inactive phases in these diets compared with RC.

### Diet-specific effects of NOB on CPS1 expression

**Fig. 2:**
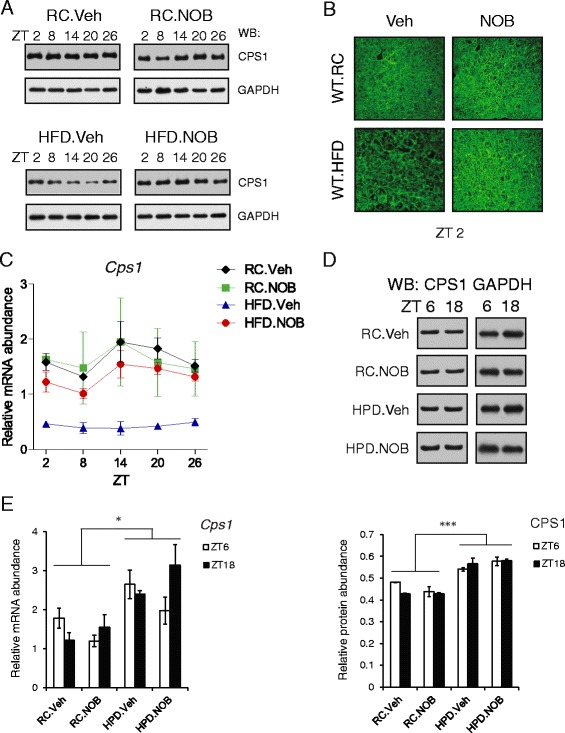
NOB modulates *Cps1* mRNA and protein expression. **a** Total protein extracts were prepared from liver samples collected from the four diet/treatment groups of wild-type mice at the indicated circadian time points (*n* = 3). Western blotting analysis was performed using anti-CPS1 antibody. *RC* regular chow, *HFD* high-fat diet, *Veh* vehicle, *NOB* Nobiletin. The results shown are representative of three independent experiments. See Additional file 1: Figure S1A for quantitative analysis. **b** Immunohistochemical staining of CPS1 in liver sections from mice with the indicated diet and treatment at ZT2. **c** Real-time RT-PCR analysis of *Cps1* in liver samples collected as in (**a**). Data are presented as mean ± SEM (*n* = 3). Two-way ANOVA with Bonferroni *post-hoc* tests shows significant statistical differences between HFD.Veh and other three groups (*p* < 0.0001). **d** Western blotting analysis of protein lysates of liver samples collected at ZT 6 and 18 from mice with the indicated diet and treatment (*n* = 3). HPD indicates high-protein diet. The images shown to the *left* are representative of three independent experiments. Quantitation of Western blots was carried out and the results, presented as mean ± SEM, are shown in the *lower* panel. Two-way ANOVA with Bonferroni *post-hoc* tests, RC vs. HPD, ***p < 0.001. **e** Real-time qPCR analysis was carried out using total RNAs extracted from the liver samples described in (**d**). The results are presented as mean ± SEM. Two-way ANOVA with Bonferroni *post-hoc* tests, RC vs. HPD, *p < 0.05

Carbamoyl phosphate synthetase I (CPS1) has been shown to accumulate in a circadian manner; however, CPS1 proteins are exceedingly abundant and discrepancy in circadian pattern exists [26, 27]. We therefore conducted Western blotting to examine CPS1 protein levels in mouse liver over circadian time course. While largely unchanged between Veh and NOB treatment under RC conditions based on 2-way ANOVA, CPS1 levels were significantly reduced in HFD.Veh compared with RC (Fig. 2a and Additional file 1: Figure S1A) [47]. Strikingly, NOB restored CPS1 in HFD to RC levels (Fig. 2a). We also confirmed the above results using Coomassie staining followed by mass spectrometry (Additional file 1: Figure S1B and Table S3). Furthermore, confocal microscopy using anti-CPS1 antibody also clearly showed recovered CPS1 in livers from HFD.NOB mice relative to HFD.Veh (Fig. 2b and Additional file 1: Figure S1C). Real-time qPCR analysis further revealed strong repression of *Cps1* messenger levels by HFD relative to RC (Fig. 2c), consistent with its effect on CPS1 proteins. Importantly, NOB restored *Cps1* mRNA expression, mirroring the changes in CPS1 protein.

We also examined effects of HPD on CPS1 levels. HPD enhanced the abundance of CPS1 by approximately 20 % (Fig. 2d), suggesting a role of CPS1 induction to cope with increased dietary protein intake. HPD feeding exerted similar modest inducing effects on *Cps1* mRNA compared with RC (Fig. 2e). NOB treatment, on the other hand, did not further increase CPS1 protein level under HPD (Fig. 2d), yet seemed to alter the mRNA expression with a slight increase at ZT18 (Fig. 2e). These results indicated opposing effects of HFD and HPD on *Cps1* expression, suggesting that ammonia-lowering activities of NOB entail diet-specific CPS1 regulatory mechanisms.

### NOB restored levels of C/EBP transcription factors repressed under HFD

**Fig. 3:**
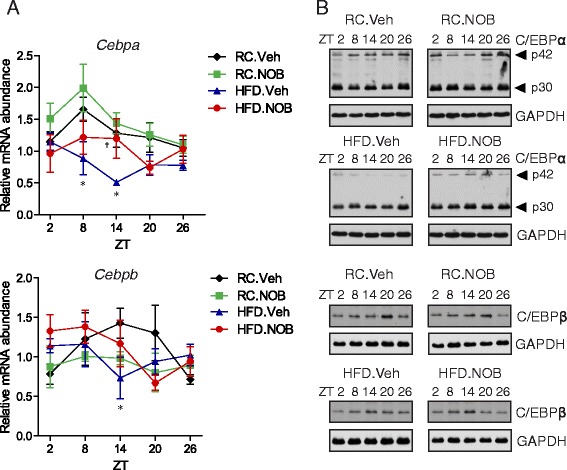
NOB rescued *Cebpa* and *Cebpb* mRNA and protein circadian expression in the liver from HFD fed mice. **a** Real-time RT-PCR analysis of *Cebpa* and *Cebpb* in livers collected at the indicated circadian times from mice with the indicated diet and treatment as in Fig. 2a. Student’s *t*-test: RC.Veh vs. HFD.Veh, *p < 0.05; HFD.Veh vs. HFD.NOB, †*p* < 0.05. **b** Western blotting was performed using total liver protein lysates with the indicated antibodies. The results are representative of three independent experiments. See Additional file 1: Figure S2 for quantitative analysis

We next focused on HFD where the above results indicated NOB-mediated transcriptional induction of *Cps1*. The transcription factors C/EBPα and C/EBPβ are known to play important roles in energy homeostasis and urea cycle expression [7, 48, 49]. Consistent with previous results [50], liver expression of both *Cebp* genes exhibited clear circadian oscillation in RC; somewhat surprisingly, NOB moderately elevated *Cebpa* expression, yet strongly repressed *Cebpb* (Fig. 3a). HFD significantly dampened levels and circadian amplitude (peak/trough difference) of both *Cebp* mRNA expression, and interestingly also reversed their circadian phase. NOB treatment largely restored *Cebpa* mRNA expression (ZT14) and circadian phase relative to RC (Fig. 3a).

HFD also significantly down-regulated protein levels of C/EBPα p42, the active form [51], across the circadian cycle (HFD.Veh vs. RC.Veh; Fig. 3b and Additional file 1: Figure S2A). In comparison, the amount of p30, the truncated form lacking the N-terminal transactivation domain, was also reduced, albeit to a lesser degree (Fig. 3b and Additional file 1: Figure S2B). Compared with Veh, NOB did not show significant effects on p42/p30 levels in RC, yet appeared to reverse their reduction by HFD to RC levels (Fig. 3b and Additional file 1: Figure S2A). HFD was previously reported to increase C/EBPβ (LAP and LIP) levels in mouse liver [52]. In comparison, our circadian analysis revealed a C/EBPβ (LAP) phase shift in HFD (Fig. 3b and Additional file 1: Figure S2C). Whereas both RC.Veh and RC.NOB showed peak C/EBPβ expression at ZT20, HFD shifted the peak to ZT14, with HFD.NOB displaying a slight increase over HFD.Veh (Fig. 3b and Additional file 1: Figure S2C). These results illustrated circadian- and diet-dependent expression patterns of C/EBPs and a novel function of NOB in restoring C/EBP levels repressed by HFD.

### C/EBP site mutations on the Cps1 promoter abolished NOB effects

**Fig. 4:**
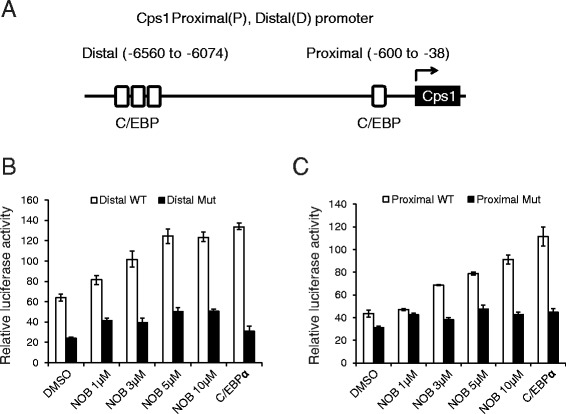
C/EBP binding site mutations in proximal and distal *Cps1* enhancer regions impaired NOB-mediated reporter activation. **a** Diagram of distal and proximal C/EBP sites on the *Cps1* promoter region. **b** and **c** Hepa1-6 cells were transfected with reporter constructs containing wild-type (WT; *white bars*) and C/EBP binding site mutated (*black bars*) enhancer regions, and treated with NOB at the indicated doses. Co-transfection of C/EBPα expression construct was also carried out in parallel. Each value represented mean ± SEM of three replicates from a single assay. The results are representative of at least three independent experiments. **b** and **c** show results from reporter constructs containing distal and proximal WT and mutant enhancers respectively. Two-way ANOVA with Bonferroni *post-hoc* tests: significant main effects of plasmid construct, (b) *F* = 81.43, *p* < 0.001, (c) *F* = 254.97, *p* < 0.0001; NOB concentration effect, (b) *F* = 47.39, *p* < 0.0001, (c) *F* = 42.01, *p* < 0.0001; and a significant interaction between constructs and NOB concentration, (b) *F* = 23, *p* < 0.0001, (c) *F* = 24.37, *p* < 0.0001

Previous studies have identified both proximal and distal C/EBP sites in the *Cps1* promoter [16, 35, 53]. We next generated reporter constructs containing either the intact *Cps1* promoter or mutant promoters respectively deficient in either C/EBP sites (Fig. 4a). NOB was found to dose-dependently activate reporter expression from the WT construct with an intact promoter (Fig. 4b and c). Distal mutations markedly repressed the reporter expression, yet seemed to at least partially retain NOB response as NOB increased mutant reporter expression relative to DMSO (Fig. 4b). In comparison, while not significantly affecting the baseline expression level, proximal mutations abolished NOB dose-dependent induction seen in the WT construct (Fig. 4c). C/EBPα co-transfection activated both proximal and distal WT reporter expression, but showed no effects on mutant constructs in the absence of NOB. These results suggested that the proximal C/EBP consensus site plays a major role in mediating the NOB induction of *Cps1*.

### Transcriptional regulation of other urea cycle genes by NOB under HFD

**Fig. 5:**
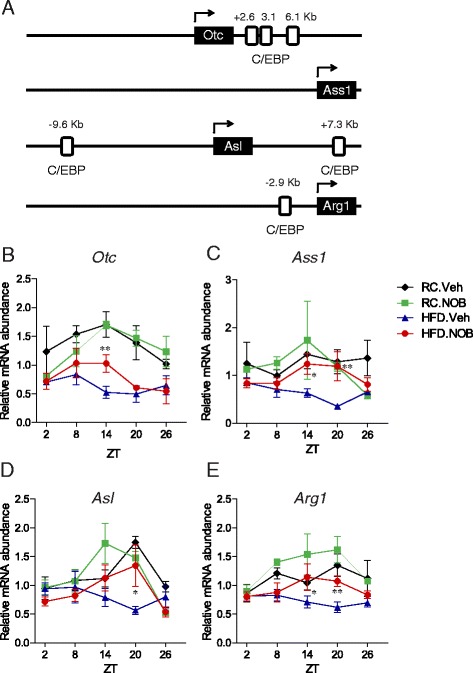
NOB coordinately enhanced urea cycle gene expression. **a** Diagram of C/EBP sites on the genomic regions for urea cycle genes including *Otc*, *Ass1*, *Asl*, and *Arg1*. **b**-**e** Liver samples were collected from the four diet/treatment groups of wild-type mice at the indicated circadian time points. Total RNAs were purified for real-time RT-PCR analysis using primers for the four urea cycle genes, including *Otc* (**b**), *Ass1* **c**, *Asl* **d** and *Arg1* **e**. *RC* regular chow, *HFD* high-fat diet, *Veh* vehicle, *NOB* Nobiletin. Data are presented as mean ± SEM (*n* = 3). Two-way ANOVA with Bonferroni *post-hoc* tests, RC.Veh vs. HFD.Veh, **a** *p* < 0.0001, **b** *p* < 0.01, **c** *p* < 0.05, **d** *p* < 0.01. Student’s *t*-test, HFD.Veh vs. HFD.NOB, *p < 0.05, **p < 0.01

A number of pioneering studies have provided strong evidence for co-regulation of urea cycle genes by diets, hormones, cAMP and other factors, likely as an evolutionary strategy to efficiently perform ureagenesis in response to various internal and external cues [8, 20, 35, 54–56]. In particular, C/EBP sites are found on several urea cycle genes (Fig. 5a) [7, 8]. Under RC, NOB treatment showed only modest effects on expression of the four other urea cycle genes (*Otc*, *Ass1*, *Asl*, *Arg1*) relative to Veh controls (Fig. 5b-e). Strikingly, whereas HFD generally repressed their mRNA levels and also elicited strong phase shifts (Fig. 5), NOB enhanced their expression and/or amplitude, and interestingly also correct circadian phases. Note that a putative C/EBP site on the *Ass1* promoter has not been reported, suggesting a secondary effect by C/EBP or other transcriptional mechanisms [35]. Since C/EBPs are master regulators of overall hepatic metabolic circuits including glucose and lipid metabolism, coordinate regulation of urea cycle genes by C/EBPs may enable metabolic cross-talk between nitrogen homeostasis and energy metabolism [55, 57].

### Circadian Clock^Δ19/Δ19^ mutant mice displayed attenuated CPS1 and C/EBP levels

**Fig. 6:**
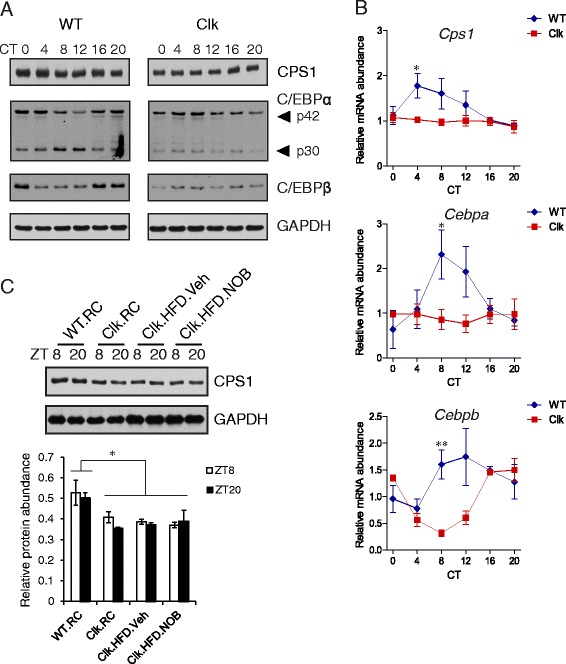
NOB restored *Cps1* and *Cebp* mRNA and protein levels in a clock-dependent manner. **a** Liver samples were collected at the indicated Circadian Times (CT) from regular chow-fed WT and *Clock* ^*Δ19/Δ19*^ mutant (Clk) mice (*n* = 3) in constant darkness (DD) when the circadian clock free runs devoid of light cue. CT0 corresponds to the onset of subjective day. Western blotting was performed using total protein extracts from the liver samples with the indicated antibodies. See Additional file 1: Figure S3 for quantitative analysis. **b** Real-time RT-PCR analysis of *Cps1, Cebpa* and *Cebpb* was carried out using total RNAs extracted from the liver samples described in (**a**)*.* Data are presented as mean ± SEM. Two-way ANOVA with Bonferroni *post-hoc* tests, WT vs. Clk, *Cps1*, *Cebpb p* < 0.01; *Cebpa p* < 0.05. Student’s *t*-test, WT vs. Clk, *Cps1*: CT4, *p < 0.05; *Cebpa*: CT8, *p < 0.05; *Cebpb*: CT8, **p < 0.01. **c** Western blot analysis of CPS1 protein levels in liver samples collected at ZT8 and ZT20 from WT and Clk mice subjected to the indicated diet and treatment. *RC* regular chow, *HFD* high-fat diet, *Veh* vehicle, *NOB* Nobiletin. Two-way ANOVA with Bonferroni *post-hoc* tests, WT vs. individual Clk groups, *p < 0.05

Consistent with previous studies [27, 50, 58], our data thus far indicated that both *Cps1* and *Cebp* genes are subjected to circadian control and display rhythmic patterns of expression. To directly examine whether the circadian clock plays an essential role in *Cps1* regulation, we employed *Clock*^*Δ19/Δ19*^ mutant mice (Clk) which express a dominant negative version of CLOCK proteins and thus are severely impaired in circadian rhythms [59, 60]. Compared with WT, CPS1, C/EBPα p30 and C/EBPβ protein levels were markedly reduced and became largely arrhythmic in Clk under RC feeding and constant darkness (DD) conditions (Fig. 6a and see also quantification in Additional file 1: Figure S3). In comparison, p42 expression and oscillation were maintained in Clk mice, yet interestingly displayed a reversed circadian phase pattern (Fig. 6a and Additional file 1: Figure S3B). These observations illustrated gene- or isoform-specific circadian expression regulation. Furthermore, pronounced expression changes in C/EBP subunits were observed in the mutant mice, including both phase shifts and reduced levels (p30 and C/EBPβ). *Cps1* and *Cebpa* mRNA expression was also compromised in the mutant mice, showing strongly reduced levels and dampened circadian amplitude (Fig. 6b). In comparison, *Cebpb* mRNA expression exhibited a marked phase shift in the mutant compared with WT (Fig. 6b, bottom), while maintaining a normal amplitude. Whereas CPS1 levels were lower in Clk than WT, neither HFD nor NOB in mutant mice conferred significant changes (Fig. 6c; lower panel: quantification). These results together indicated a critical role of a functional clock to mediate NOB induction of CPS1.

### NOB modulated serum and urine urea content

**Fig. 7:**
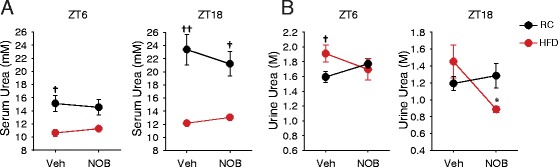
Effects of NOB on urea production. **a** and **b** Serum (**a**) and urine (**b**) urea levels at ZT6 and ZT18 in WT mice treated with Veh or NOB under RC (*n* = 7) or HFD (*n* = 4). Data are presented as mean ± SEM. Student’s *t*-test, Veh vs. NOB, *p <0.05; RC vs. HFD, †*p* <0.05, ††*p* <0.01

Finally, to further elucidate a modulatory role of NOB in nitrogen homeostasis, we measured urea content in serum and urine at both day- and night-time. In RC, serum urea levels were mildly reduced in the NOB group at both day- and night-time (ZT6 and 18) (Fig. 7a), whereas the urine urea levels showed the opposite trend, suggesting increased urine urea excretion by NOB (Fig. 7b). In comparison, HFD feeding led to lower serum urea levels than RC (Fig. 7a). More importantly, HFD showed a reverse NOB response; specifically, NOB increased serum urea and concomitantly reduced renal urea excretion, particularly at ZT18 (Fig. 7b). These results further illustrated a modulatory role of NOB in nitrogen homeostasis.

## Discussion

In the current study, we demonstrated a broad effect of the natural flavonoid NOB in reducing serum ammonia levels under varying diet conditions. NOB and other related polymethoxylated flavonoids are dietary components generally showing favorable pharmacokinetic profiles [61, 62], and mouse and human studies revealed promising efficacy of NOB against the metabolic syndrome without significant toxicity [40, 44, 45, 63]. The current work illustrated a novel function of NOB in ammonia disposal, extending the long list of physiological benefits associated with NOB and other natural flavonoids [63, 64]. In light of these beneficial attributes, it warrants further investigation whether NOB can be incorporated into dietary management for congenital and injury-induced hyperammonemia.

Our study revealed broad ammonia-lowering activities of NOB in mice fed with RC, HFD or HPD, ostensibly utilizing distinct, diet-specific mechanisms. Whereas transcriptional activation of CPS1 via C/EBP induction seems to operate under HFD, NOB treatment in conjunction with RC and HPD feeding appeared not to elicit significant CPS1 induction. Moreover, in contrast to RC where NOB similarly reduced serum ammonia at both ZT6 and ZT18, in HFD and HPD NOB more strongly diminished the relative high ammonia levels in respective diets (ZT6 for HFD and ZT18 for HPD). As a result, NOB functions to reverse the exaggerated ammonia variation between the active and inactive phases, essentially normalizing circadian ammonia rhythm in HFD and HPD to RC levels. Given the remarkably diverse cellular pathways known to be modulated by NOB and other flavonoids, various molecular and cellular mechanisms may underlie such mechanistic and functional plasticity [63–67]. Besides transcriptional regulation (C/EBP, glucocorticoid receptor, CREB and HNF3) and the allosteric cofactor NAG [7, 17], recent mechanistic studies have also underscored the importance of post-translational modifications (PTMs) for CPS1 protein activity [11–13, 47]. Other urea cycle genes may also serve as the primary target; one such candidate is *Otc*, especially given its functional connection with the circadian clock [19]. Finally, NOB may also attenuate HPD-induced bacterial production of ammonia by amino acid deamination and urea hydrolysis [4].

Our analysis revealed differential responses of serum and urine urea levels to NOB treatment in HFD. Urea represents the most predominant circulating nitrogen reservoir, and blood urea nitrogen (BUN) level is known to be regulated by multiple factors including protein intake, protein metabolism and renal function [1, 5]. HFD contains lower protein content than RC (20 % vs. 29 % in calories), and NOB acts to attenuate ammonia levels by activating urea cycle gene expression. Thus, urea may be salvaged to maintain nitrogen balance in the HFD.NOB condition. For example, urine excretion rate/volume and renal reabsorption of water/mineral/urea are tightly coupled to regulate osmotic pressure, which may in turn modulate serum and urine urea levels. However, a direct role of NOB in renal function remains to be elucidated.

Amino acid levels in human blood have been shown to exhibit robust circadian oscillation, concordant with results from a yeast metabolic cycle resembling mammalian circadian rhythm [68–70]. In parallel with fluctuating amino acid levels over the daily cycle, CPS1 and several other urea cycle components exhibited circadian mRNA and protein expression patterns [27]. However, much conflictory evidence has also been reported with regard to *Cps1* circadian expression [26, 28, 29]. Our experiments, using specific primers and antibodies, revealed weak oscillation of *Cps1* mRNA and protein. Interestingly, NOB appeared to augment CPS1 protein oscillation under RC, and also enhanced CPS1 levels under HFD in a circadian clock-dependent manner. One possible mechanism of circadian regulation may depend on C/EBPs as their expression is subjected to clock control [50], although other mechanisms likely exist [19]. Of note, we observed distinct circadian changes of C/EBPα p42 and p30 isoforms in *Clock*^*Δ19/Δ19*^ mutant mice relative to WT, suggesting a possible circadian regulation of alternative translation. Currently, work in our lab aims to further understand the detailed mechanism underlying the interaction between the clock, diet and NOB in nitrogen homeostasis.

## Conclusion

In this current study, we present physiological evidence indicating that NOB exhibits a broad activity in ammonia control in mice fed with varying diets. Molecular experiments further revealed that NOB regulates urea cycle function via C/EBP- and circadian clock-dependent regulatory mechanisms. These observations together demonstrate a novel physiological function and cellular mechanism of a natural dietary flavonoid in nitrogen homeostasis.

## Additional file

Additional file 1: Table S1. Primers for construction of reporter constructs. **Table S2.** Primers for real-time qPCR analysis. **Table S3.** Mass spectrometry data table for the 164KD band shown in the Additional file 1: Figure S1B. **Figure S1.** NOB modulates CPS1 expression. **(A)** Quantification of CPS1 protein levels from three independent experiments including the blot shown in Fig. 2a. RC, regular chow; HFD, high-fat diet; Veh, vehicle; NOB, Nobiletin. Two-way ANOVA with Bonferroni *post-hoc* tests shows significant statistical differences between HFD.Veh and other three groups (*p* < 0.0001). Furthermore, one-way ANOVA with Bonferroni tests shows significant difference (*p* < 0.05) between ZT time points in RC.Veh but not in other groups. **(B)** Coomassie blue staining of CPS1. Mouse liver protein lysates were separated on SDS-PAGE gel and stained with Coomassie blue. The predominant 164KD band was validated by mass spectrometry as CPS1 (Table S3). **(C)** Control microscopy images for Fig. 2b using rabbit IgG. **Figure S2.** NOB rescued C/EBP protein circadian expression in the liver from HFD fed mice. **(A)** Quantification of C/EBPα p42 protein levels from three independent experiments including the blot shown in Fig. 3b. Two-way ANOVA with Bonferroni post-hoc tests shows significant difference between RC.Veh and HFD.Veh (*p* < 0.05), indicating diet effect on the C/EBPα p42 expression level. Importantly, RC.NOB was not significantly different from HFD.NOB, suggesting NOB reversed the reducing effect of HFD on the C/EBPα p42 expression. **(B)** Quantification of C/EBPα p30 protein levels from three independent experiments including the blot shown in Fig. 3b. Two-way ANOVA with Bonferroni post-hoc tests shows significant difference between RC.Veh and HFD.Veh (*p* < 0.01), but RC.NOB and HFD.NOB was not significantly different, again suggesting NOB reversed the reducing effect of HFD on the p30 expression. **(C)** Quantification of C/EBPβ protein levels from three independent experiments including the blot shown in Fig. 3b. Two-way ANOVA with Bonferroni post-hoc tests shows significant difference between RC.Veh and HFD.Veh (*p* < 0.0001), but RC.NOB and HFD.NOB was not significantly different. **Figure S3.** NOB restored CPS1 and C/EBP protein levels in a clock-dependent manner. **(A)** Quantification of CPS1 protein levels under constant darkness (DD) conditions from three independent experiments including the blot shown in Fig. 6a. Two-way ANOVA with Bonferroni *post-hoc* tests shows significant statistical difference between WT and Clk (*Clock* ^*Δ19/Δ19*^) (*p* < 0.0001). **(B)** Quantification of C/EBPα p42 protein levels under constant darkness (DD) conditions from three independent experiments including the blot shown in Fig. 6a. Two-way ANOVA with Bonferroni *post-hoc* tests shows significant statistical differences between WT and Clk (*p* < 0.05). **(C)** Quantification of C/EBPα p30 protein levels under constant darkness (DD) conditions from three independent experiments including the blot shown in Fig. 6a. Two-way ANOVA with Bonferroni *post-hoc* tests shows significant statistical difference between WT and Clk (*p* < 0.01). **(D)** Quantification of C/EBPβ protein levels under constant darkness (DD) conditions from three independent experiments including the blot shown in Fig. 6a. Two-way ANOVA with Bonferroni *post-hoc* tests shows significant statistical difference between WT and Clk (*p* < 0.05).

## Abbreviations

CPS1: Carbamoyl phosphate synthetase 1
RC: Regular chow
HFD: High-fat diet
Veh: Vehicle
NOB: Nobiletin
HPD: High-protein diet
NAG: N-acetylglutamate
ZT: Zeitgeber time
OTC: Ornithine transcarbamylase
C/EBP: CCAAT/enhancer binding protein
WT: Wild-type
Clk: *Clock*^*Δ19/Δ19*^ mutant
PTMs: Post-translational modifications
