# Supplementary material for: Ammonia-lowering activities and carbamoyl phosphate synthetase 1 (Cps1) induction mechanism of a natural flavonoid
Source: Nutr Metab (Lond). 2015 Jun 9;12:23. doi: 10.1186/s12986-015-0020-7 (PMC4465466; doi:10.1186/s12986-015-0020-7)
Supplement: Additional file 1: Table S1. — Primers for construction of reporter constructs. Table S2. Primers for real-time qPCR analysis. Table S3. Mass spectrometry data table for the 164KD band shown in the Additional file 1: Figure S1B. Figure S1. NOB modulates CPS1 expression. (A) Quantification of CPS1 protein levels from three independent experiments including the blot shown in Fig. 2a. RC, regular chow; HFD, high-fat diet; Veh, vehicle; NOB, Nobiletin. Two-way ANOVA with Bonferroni post-hoc tests shows significant statistical differences between HFD.Veh and other three groups (p < 0.0001). Furthermore, one-way ANOVA with Bonferroni tests shows significant difference (p < 0.05) between ZT time points in RC.Veh but not in other groups. (B) Coomassie blue staining of CPS1. Mouse liver protein lysates were separated on SDS-PAGE gel and stained with Coomassie blue. The predominant 164KD band was validated by mass spectrometry as CPS1 (Table S3). (C) Control microscopy images for Fig. 2b using rabbit IgG. Figure S2. NOB rescued C/EBP protein circadian expression in the liver from HFD fed mice. (A) Quantification of C/EBPα p42 protein levels from three independent experiments including the blot shown in Fig. 3b. Two-way ANOVA with Bonferroni post-hoc tests shows significant difference between RC.Veh and HFD.Veh (p < 0.05), indicating diet effect on the C/EBPα p42 expression level. Importantly, RC.NOB was not significantly different from HFD.NOB, suggesting NOB reversed the reducing effect of HFD on the C/EBPα p42 expression. (B) Quantification of C/EBPα p30 protein levels from three independent experiments including the blot shown in Fig. 3b. Two-way ANOVA with Bonferroni post-hoc tests shows significant difference between RC.Veh and HFD.Veh (p < 0.01), but RC.NOB and HFD.NOB was not significantly different, again suggesting NOB reversed the reducing effect of HFD on the p30 expression. (C) Quantification of C/EBPβ protein levels from three independent experiments including the blot shown in Fig. 3b. Two-wa [file 12986_2015_20_MOESM1_ESM.pdf]

Additional File

Including Supplementary Tables S1-3, Supplementary figure legends and Supplementary Figures S1-3

Ammonia-lowering activities and carbamoyl phosphate synthetase 1 (Cps1) induction mechanism of a natural flavonoid

Kazunari Nohara[1a], Youngmin Shin[1a], Noheon Park[2], Kwon Jeong[1], Baokun He[1], Nobuya Koike[3], Seung-Hee Yoo[1], Zheng Chen[1*]

[1]Department of Biochemistry and Molecular Biology, The University of Texas Health Science Center at Houston, Houston, TX 77030, USA; [2]Department of Neuroscience, The University of Texas Southwestern Medical Center, Dallas, TX 75390, USA; [3]Department of Physiology and Systems Bioscience, Kyoto Prefectural University of Medicine, Kyoto, 602-8566, Japan.

[a]These authors contributed equally.

Running title: Ammonia control and urea cycle regulation by a flavonoid

*Corresponding author: Zheng Chen, Department of Biochemistry and Molecular Biology, The University of Texas Health Science Center at Houston, 6431 Fannin Street, MSB 6.200, Houston, TX 77030, USA. Tel.: 713-500-6284; Fax: 713-500-0652; E-mail: zheng.chen.1@uth.tmc.edu .

Supplementary Table S1: Primers for construction of reporter constructs.

**Name**	**Sequence**
pF	5'-GGCCCCGGGAGATCTGGAGATACACAGTAAATT-3'
pR	5'-GGCCCATGGCAGCTCCTCCTTTCCTTAGCCCCT-3'
P-C/EBPmutF	5'-GAGAAGGTGCCACTTGTTATATTATGATTTGTATGACATGTCCATT-3'
P-C/EBPmutR	5'-AATGGACATGTCATACAAATCATAATATAACAAGTGGCACCTTCTC-3'
dF	5'-GGCCCCGGGCAGTTTCCGAGAATCTGAAACACA-3'
dR	5'-GGCCCATGGTTTGAAAACAGCAAATTCATCAGC-3'

Supplementary Table S2: Primers for real-time qPCR analysis.

**Target genes**	**Forward primer**	**Reverse primer**
GAPDH	5'-CAAGGTCATCCATGACAACTTTG-3'	5'-GGCCATCCACAGTCTTCTGG-3'
Cps1	5'-CACCAATTTCCAGGTGACCA-3'	5'-TACTGCTTTAGGCGGCCTTT-3'
Otc	5'-AGGGTCACACTTCTGTGGTTC-3'	5'-CAGAGAGCCATAGCATGTACTG-3'
Ass1	5'-ACACCTCCTGCATCCTCGT-3'	5'-GCTCACATCCTCAATGAACACCT-3'
Asl	5'-CTATGACCGGCATCTGTGGAA-3'	5'-AGCAACCTTGTCCAACCCTTG-3'
Arg1	5'-TTGGGTGGATGCTCACACTG-3'	5'-GTACACGATGTCTTTGGCAGA-3'
Cebpa	5'-CAAGAACAGCAACGAGTACCG-3'	5'-GTCACTGGTCAACTCCAGCAC-3'
Cebpb	5'- ACCGGGTTTCGGGACTTGA -3'	5'- GTTGCGTAGTCCCGTGTCCA -3'

Supplementary Table S3: Mass spectrometry data table for the 164KD band shown in the Supplemental Figure 1B.

Accession	Description	Score	Coverage	Unique Peptides	PSMs	MW [kDa]
124248512	carbamoyl-phosphate synthetase [ammonia], mitochondrial precursor [Mus musculus]	13654.31	66.47	106	778	164.5
124486747	glycogen debranching enzyme [Mus musculus]	1273.25	36.88	54	85	174.2
146219837	eukaryotic translation initiation factor 3 subunit A [Mus musculus]	870.20	35.04	49	69	161.8
19527028	vigilin [Mus musculus]	1147.90	38.17	45	78	141.7
93102409	fatty acid synthase [Mus musculus]	614.67	13.50	29	31	272.3
110347469	alpha-2-macroglobulin precursor [Mus musculus]	631.93	23.68	27	47	165.7
114205420	aldehyde oxidase 3 [Mus musculus]	862.84	21.95	25	37	146.8
77682555	xanthine dehydrogenase/oxidase [Mus musculus]	704.11	19.70	25	39	146.5
254553372	isoleucine--tRNA ligase, cytoplasmic [Mus musculus]	342.94	22.98	25	28	144.2

Supplementary figure legends:

Supplementary Figure S1. NOB modulates CPS1 expression. (A) Quantification of CPS1 protein levels from three independent experiments including the blot shown in Fig. 2A. RC, regular chow; HFD, high-fat diet; Veh, vehicle; NOB, Nobiletin. Two-way ANOVA with Bonferroni *post-hoc* tests shows significant statistical differences between HFD.Veh and other three groups (p<0.0001). Furthermore, one-way ANOVA with Bonferroni tests shows significant difference (p<0.05) between ZT time points in RC.Veh but not in other groups. **(B)** Coomassie blue staining of CPS1. Mouse liver protein lysates were separated on SDS-PAGE gel and stained with Coomassie blue. The predominant 164KD band was validated by mass spectrometry as CPS1 (Table S3). **(C)** Control microscopy images for Figure 2B using rabbit IgG.

Supplementary Figure S2. NOB rescued C/EBP protein circadian expression in the liver from HFD fed mice. (A) Quantification of C/EBPα p42 protein levels from three independent experiments including the blot shown in Figure 3B. Two-way ANOVA with Bonferroni *post-hoc* tests shows significant difference between RC.Veh and HFD.Veh (p<0.05), indicating diet effect on the C/EBPα p42 expression level. Importantly, RC.NOB was not significantly different from HFD.NOB, suggesting NOB reversed the reducing effect of HFD on the C/EBPα p42 expression. **(B)** Quantification of C/EBPα p30 protein levels from three independent experiments including the blot shown in Figure 3B. Two-way ANOVA with Bonferroni *post-hoc* tests shows significant difference between RC.Veh and HFD.Veh (p<0.01), but RC.NOB and HFD.NOB was not significantly different, again suggesting NOB reversed the reducing effect of HFD on the p30 expression. **(C)** Quantification of C/EBPβ protein levels from three independent experiments including the blot shown in Figure 3B. Two-way ANOVA with Bonferroni *post-hoc* tests shows significant difference between RC.Veh and HFD.Veh (p<0.0001), but RC.NOB and HFD.NOB was not significantly different.

Supplementary Figure S3. NOB restored CPS1 and C/EBP protein levels in a clock-dependent manner. **(A)** Quantification of CPS1 protein levels under constant darkness (DD) conditions from three independent experiments including the blot shown in Figure 6A. Two-way ANOVA with Bonferroni *post-hoc* tests shows significant statistical difference between WT and Clk ($Clock^{\Delta 19/\Delta 19}$) ($p$<0.0001). **(B)** Quantification of C/EBPα p42 protein levels under constant darkness (DD) conditions from three independent experiments including the blot shown in Figure 6A. Two-way ANOVA with Bonferroni *post-hoc* tests shows significant statistical differences between WT and Clk (p<0.05). **(C)** Quantification of C/EBPα p30 protein levels under constant darkness (DD) conditions from three independent experiments including the blot shown in Figure 6A. Two-way ANOVA with Bonferroni *post-hoc* tests shows significant statistical difference between WT and Clk (p<0.01). **(D)** Quantification of C/EBPβ protein levels under constant darkness (DD) conditions from three independent experiments including the blot shown in Figure 6A. Two-way ANOVA with Bonferroni *post-hoc* tests shows significant statistical difference between WT and Clk (p<0.05).

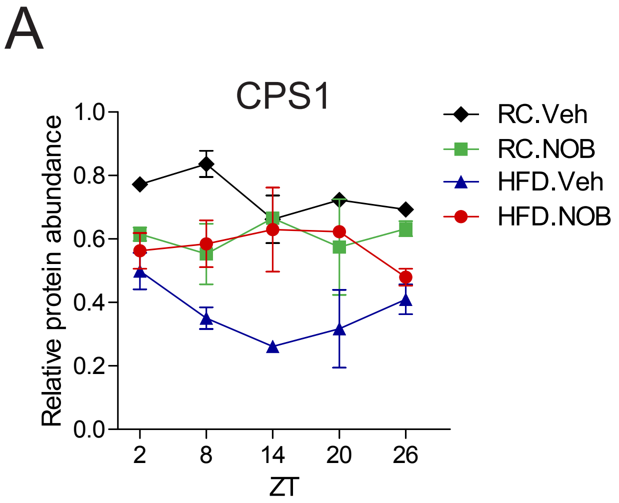
A
CPS1
Relative protein abundance
1.0
0.8
0.6
0.4
0.2
0.0
2
8
14
20
26
ZT
RC.Veh
RC.NOB
HFD.Veh
HFD.NOB

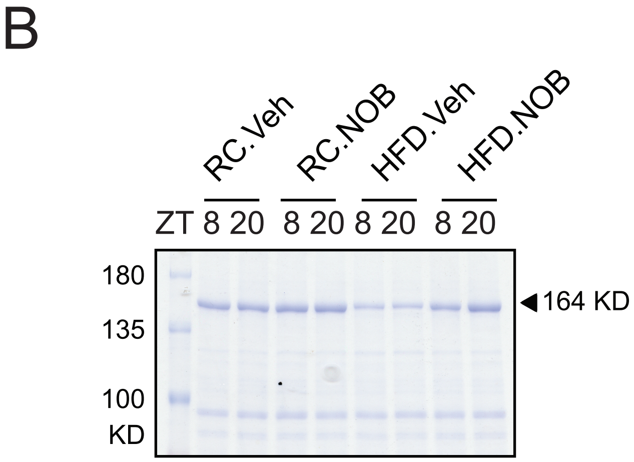
B
RC.Veh
RC.NOB
HFD.Veh
HFD.NOB
ZT 8 20 8 20 8 20 8 20
180
135
100
KD
164 KD

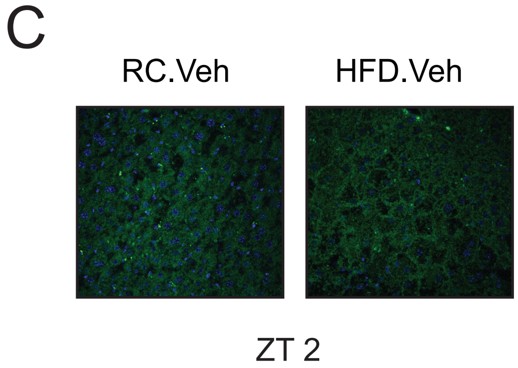
C
RC.Veh
HFD.Veh
ZT 2

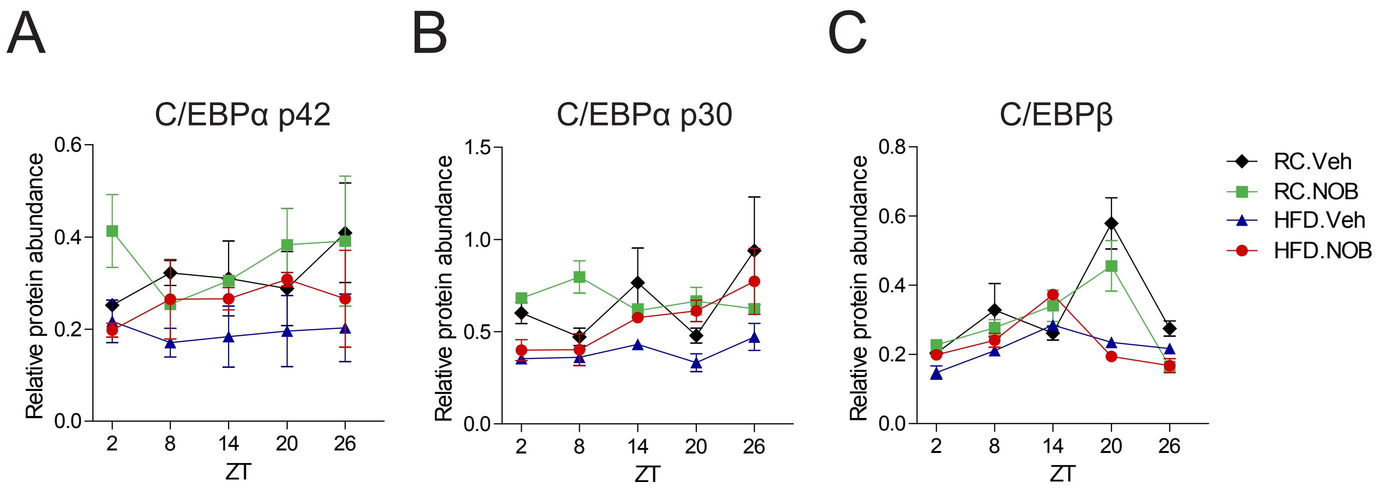
A
C/EBPα p42
Relative protein abundance
0.0
0.2
0.4
0.6
2
8
14
20
26
ZT
B
C/EBPα p30
Relative protein abundance
0.0
0.5
1.0
1.5
2
8
14
20
26
ZT
C
C/EBPβ
Relative protein abundance
0.0
0.2
0.4
0.6
0.8
2
8
14
20
26
ZT
RC.Veh
RC.NOB
HFD.Veh
HFD.NOB

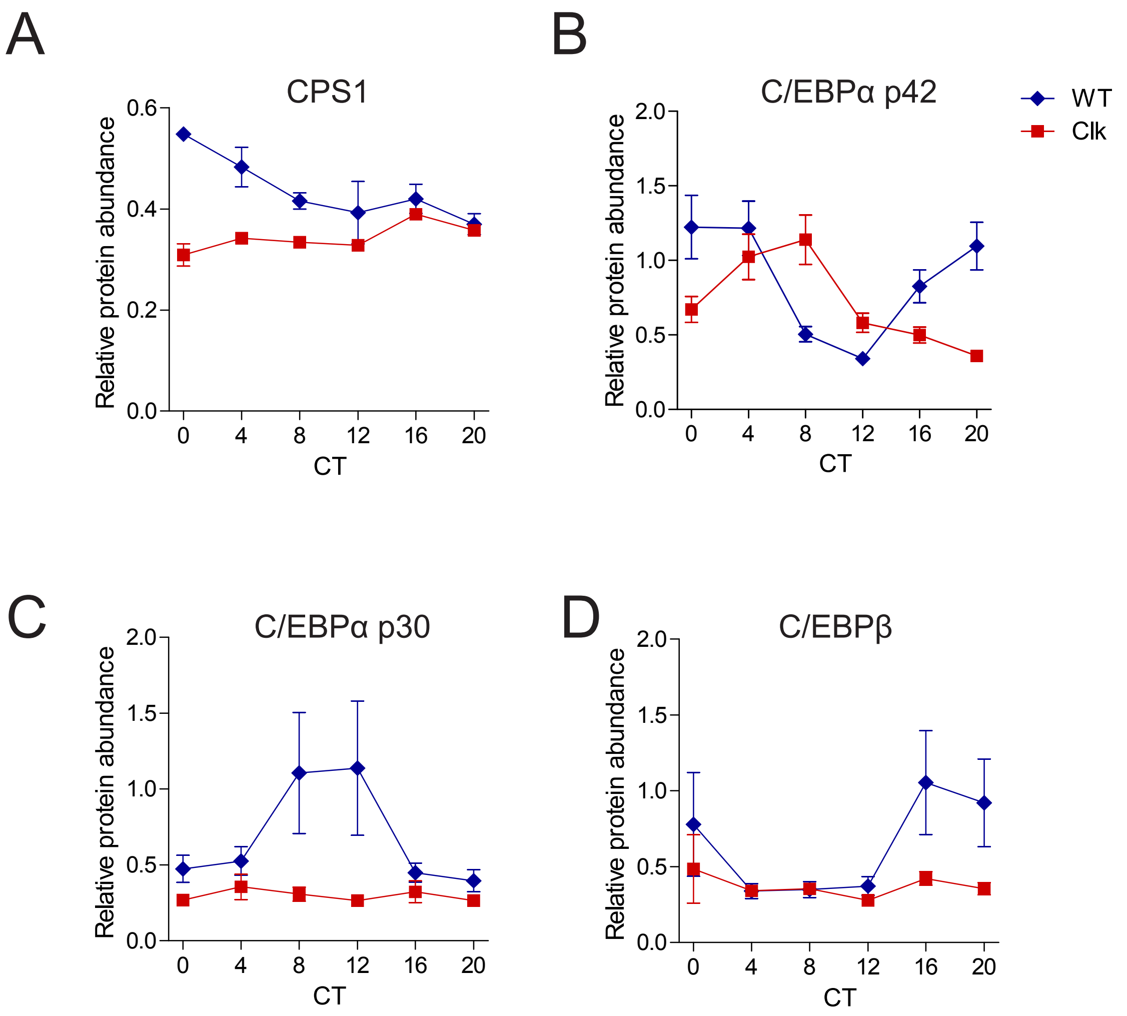
A
CPS1
Relative protein abundance
0.0
0.2
0.4
0.6
0
4
8
12
16
20
CT
B
C/EBPα p42
WT
Clk
Relative protein abundance
0.0
0.5
1.0
1.5
2.0
CT
C
C/EBPα p30
Relative protein abundance
CT
D
C/EBPβ
Relative protein abundance
CT
